# Impact of genetic predisposition to late-onset neurodegenerative diseases on early life outcomes and brain structure

**DOI:** 10.1038/s41398-024-02898-9

**Published:** 2024-04-11

**Authors:** Natalia S. Ogonowski, Luis M. García-Marín, Amali S. Fernando, Victor Flores-Ocampo, Miguel E. Rentería

**Affiliations:** 1 Mental Health & Neuroscience Program, QIMR Berghofer Medical Research Institute, Brisbane, QLD Australia; 2 School of Biomedical Sciences, Faculty of Medicine, The University of Queensland, Brisbane, QLD Australia

**Keywords:** Personalized medicine, Predictive markers

## Abstract

Most patients with late-onset neurodegenerative diseases such as Alzheimer’s and Parkinson’s have a complex aetiology resulting from numerous genetic risk variants of small effects located across the genome, environmental factors, and the interaction between genes and environment. Over the last decade, genome-wide association studies (GWAS) and post-GWAS analyses have shed light on the polygenic architecture of these diseases, enabling polygenic risk scores (PRS) to estimate an individual’s relative genetic liability for presenting with the disease. PRS can screen and stratify individuals based on their genetic risk, potentially years or even decades before the onset of clinical symptoms. An emerging body of evidence from various research studies suggests that genetic susceptibility to late-onset neurodegenerative diseases might impact early life outcomes, including cognitive function, brain structure and function, and behaviour. This article summarises recent findings exploring the potential impact of genetic susceptibility to neurodegenerative diseases on early life outcomes. A better understanding of the impact of genetic susceptibility to neurodegenerative diseases early in life could be valuable in disease screening, detection, and prevention and in informing treatment strategies before significant neural damage has occurred. However, ongoing studies have limitations. Overall, our review found several studies focused on *APOE* haplotypes and Alzheimer’s risk, but a limited number of studies leveraging polygenic risk scores or focused on genetic susceptibility to other late-onset conditions.

## Introduction

Neurodegenerative diseases (NDDs) are characterised by the selective and progressive death of neuron populations in specific brain regions, typically occurring in adults [1]. Parkinson’s disease (PD), motor neuron diseases (MND), multiple system atrophy (MSA), Alzheimer’s disease (AD), and frontotemporal dementia (FTD) are among the most common types of NDDs [2]. Although NDDs are classified primarily based on their clinical features, the molecular hallmarks of neural loss and their anatomic distribution are often used to identify similarities and differences in their nosology. For example, PD is characterised by the progressive loss of dopaminergic neurons in the *substantia nigra*, and the appearance of intracellular inclusions called Lewy bodies [3]. In contrast, the molecular pathogenesis of AD is primarily driven by the accumulation of neurofibrillary tangles and amyloid plaques in the entorhinal cortex, hippocampus, and basal forebrain [4].

While familial or monogenic mutations in specific genes directly influence metabolic dysfunction, leading to amyloidosis, tauopathies, TDP-43 proteinopathies, and α-synucleinopathies, NDDs can also be idiopathic or complex. Complex polygenic forms of NDDs result from intricate interactions between the effects of environmental factors and hundreds or thousands of genetic risk variants exerting small individual effects [5]. Genome-wide association studies (GWAS) are commonly used to study complex forms of NDDs, enabling the mapping of specific genetic markers. Post-GWAS analyses can leverage this data to identify high-risk individuals, which could help develop novel prevention and treatment strategies [5, 6].

Polygenic risk scores (PRS) estimate an individual’s lifetime genetic risk for a given phenotype [7]. PRS could help investigate individuals with a high genetic liability for a specific condition, which puts them at high risk for developing the disease. Risk can be ascertained years or decades before the expected onset of clinical symptoms. Thus, early implementation of PRS could be valuable in informing the diagnosis and personalised treatment strategies for NDDs.

The prevalence of the two most common NDDs, AD and PD, is ~5–8% and 2–3% of the worldwide population over 60 years of age, respectively [8–11]. The number of people diagnosed with NDDs is expected to triple by 2050 due to population ageing, posing substantial challenges to the healthcare system, economy, and society [12]. Therefore, understanding the influences of genetic susceptibility to these conditions in early life, before overt symptoms manifest, is critical in developing successful screening programs and interventions. Studies investigating NDDs can now investigate the effect of genetic risk factors at different stages of life, including infants, children, adolescents, young adults, and adults, and their role in the onset and progression of NDDs. This review aims to synthesise the current understanding of the association between genetic factors associated with NDDs and phenotypes such as cognitive function, brain structure and function, and behaviour in different age groups. We also discuss the limitations of current research on this topic and present potential research avenues that future studies could explore.

## Alzheimer’s disease

AD impairs cognitive function and is linked to changes in brain structure and function. While the ε4 allele of the apolipoprotein E (*APOE4*) gene is a well-known genetic risk factor for the development of sporadic AD, recent GWAS studies have uncovered dozens of other genetic risk factors, opening the doors to studying the potential impact of additive genetic risk for AD on early life.

### Effects of AD genetic risk factors on cognitive function in early life

#### *APOE* haplotype

Cognitive decline trajectories likely result from the additive effects of genetic and non-genetic factors over the life course. Several studies have focused on the association between *APOE* ε4 and cognitive decline in healthy individuals during early life [13, 14]. *APOE* ε4 carriers without dementia exhibit, on average, a more rapid decline in memory, processing speed, and language functions than non-carriers before the age of 60 [15, 16]. Furthermore, research has shown that healthy ε4 carriers are more likely to experience slight detriments in educational attainment and IQ performance from childhood to adolescence compared to non-*APOE* ε4 carriers [17–21]. Interestingly, Reynolds et al. reported that *APOE* ε4-associated cognitive decline was higher in healthy females than healthy males between 6 and 18 years of age, suggesting that differential risks for AD in females might emerge in earlier stages of development, particularly for reasoning traits [22]. Additionally, some studies have observed an association between *APOE* ε4 in healthy females among children, adolescents and young adults and lower IQ, suggesting that this effect may be more pronounced with increasing age and in certain sex-specific populations [23, 24].

Understanding the impact of genetic factors on long-term cognitive trajectories is challenging due to the high heterogeneity of these associations and the disparities in rates of cognitive decline in early life. Although some studies suggest that patterns of cognitive changes associated with AD-genetic risk profiles might be observed early in life, others argue for a broader perspective, suggesting that cognitive function is not significantly affected in healthy *APOE* ε4 carriers [25–27]. However, it should be noted that other studies have adopted a different perspective, suggesting that cognitive function may be better in healthy *APOE* ε4 carriers [28, 29]. Further research is needed to reconcile these conflicting findings and provide a comprehensive understanding of the impact of genetic factors on cognitive trajectories in early life.

Some studies have reported a slightly lower cognitive function in young *ε4* carriers [30–33], while others have reported no difference or even higher cognitive performance in young ε4 carriers [34–37]. Following these observations, the *antagonistic pleiotropy hypothesis* of *APOE* ε4 has been proposed, where individual loci/alleles have different effects on cognitive function throughout the lifespan [38, 39]. This hypothesis suggests that a lower or null negative effect may be observed during childhood and adolescence [40], while an increased positive effect may be seen in adulthood [35, 37], with significantly higher detrimental effects observed among the elderly [41]. However, understanding the complex relationship between APOE and cognitive function in early life remains controversial.

However, several aspects of the antagonistic pleiotropy hypothesis of *APOE* have been questioned. Two meta-analyses examined associations between seven cognitive domains and functional differences in executive-frontal neural networks in younger ε4 and non-ε4 carriers. However, they did not find evidence to support the ε4 allele as a pleiotropic gene [42, 43]. Taken together, these studies showed that the impact of *APOE* genotypes on cognitive function in healthy subjects might represent a complex pattern in which exposure to adverse non-genetic factors - such as educational attainment, IQ, sex, ethnicity, and age - from early in development to late life result in diverse outcomes. Therefore, further studies investigating the effect of *APOE* on cognition at different stages of life in healthy individuals are required.

#### AD polygenic risk scores

Combining the additive effects of common genetic risk variants into a PRS can enable individual stratification based on their AD risk beyond the *APOE* genotype [44]. Recent studies have suggested that cognitive functions might be affected by AD’s polygenic risk scores (AD-PRS) in early life [33, 45, 46] study conducted in a cohort of United Kingdom children revealed a relationship between higher AD-PRS (including *APOE*), lower IQ, and poorer academic achievement. However, removing genetic variants related to educational attainment attenuated this association, which might indicate the presence of vertical pleiotropic effects [47]. Recent research has shown that the association between AD-PRS and educational attainment might vary depending on the number of *APOE* ε4 alleles, with a stronger effect observed in individuals who carry two copies of the allele. Although there is evidence that educational attainment could attenuate the effect of the *APOE* ε4 allele on brain pathologies with ageing, the biological mechanisms underlying the effects are still unknown.

In addition, a study including two cohorts of Brazilian children aged 8–14 years, with a discovery sample from Porto Alegre (*N* = 364) and a replication sample from Sao Paulo (*N* = 352), revealed an association between increased AD-PRS (including *APOE*) and detriment in non-declarative memory tasks performance in *APOE* ε3/ε3 carriers, suggesting that cognitive differences are not solely driven by the *APOE* ε4 allele [45]. Moreover, Axelrud et al. observed that AD-PRS (including *APOE*) might impact brain connectivity between the right precuneus and the right superior temporal gyrus, influencing immediate and delayed recall [33, 45]. Notably, these regions have been reported to be affected by the accumulation of tau protein, as identified in studies by Hoenig et al. In fact, Hoeging et al. identified ten independently coherent tau pathology networks in AD that were associated with disease progression. They coincided with highly functionally connected brain regions such as the precuneus and cingulate cortex [48]. However, caution is needed in interpreting causal relationships from these associations and drawing conclusions about the pathways of tau spread through functional networks, as tau deposition may vary across patients and disease stages, and other factors such as age and amyloid-β may also play a role in this process [49].

Verhaaren et al. reported an association of the AD-PRS (including *APOE*) with global cognition, memory, and processing speed in young adults and elderly non-dementia subjects. However, this correlation was attenuated by excluding *APOE* ε4 from the risk score, which suggests ε4 carriers might experience detrimental effects on cognition regardless of any pathological changes related to AD [46]. Similar findings were observed by Mormino et al., who reported higher AD-PRS and AD-like ß-amyloid protein levels in 1322 healthy younger participants aged 18–33 years. It is noted that this study used a less stringent significance level inclusion criterion for SNPs in the PRS, including SNPs below the standard GWAS p-value threshold (*p* = 5 × 10^−08^) [50].

However, the estimation of PRS can be influenced by various factors, including differences in cognitive measures employed, sample demographics, statistical power, inclusion and exclusion criteria of the *APOE* genotype, and allelic heterogeneity across samples. These factors may contribute to inconsistencies in the findings of previous studies where an attenuated or null effect of PRS on healthy cohorts in early life was observed. In addition, Lamballais et al. reported no significant association between the genetic burden for AD and cognitive performance and change during childhood [51]. Similarly, other studies did not find evidence for an association between cognitive performance in children and AD-PRS or *APOE*, suggesting that any detrimental effects of AD-associated genetic risk on cognitive function are not meaningful until later in life [27, 47].

Despite the observed attenuated AD-PRS in some studies, consolidating both *APOE* haplotypes and PRS alongside other risk factors may enable more accurate risk prediction [52, 53]. The *APOE* ε4 allele has been implicated in neuropathological processes such as tau pathology and inflammation. However, whether and how *APOE* ε4 affects brain health in non-dementia aging is unknown. Furthermore, much uncertainty remains about how these associations develop in children long before disease onset. Nevertheless, limited available studies suggest that these associations may have roots in neurodevelopment, which could open up opportunities for AD risk detection and intervention long before the onset of the disease [45]. These advances might facilitate the development of new therapies and provide insights into how genetic differences impact cognitive decline progression in both early and late life.

### Effects of AD genetic risk factors on brain structure and function in early life

#### *APOE* haplotype

Recent research has delved into the impact of *APOE* and AD polygenic risk on brain structure using magnetic resonance imaging (MRI). Brain development depends on intricate mechanisms such as the functional specialisation of grey matter regions and the myelination of white matter connections between neuronal networks. Notably, it has been hypothesised that individuals with higher AD risk, particularly *APOE* ε4 carriers, may experience brain changes during childhood or early adulthood, well before the accumulation of β-amyloid in the brain. This suggests that AD genetic risk may affect postnatal neurodevelopment during early life.

Large cross-sectional imaging studies have confirmed significant differences in brain volume, fractional anisotropy, and thinning in healthy children and young individuals, particularly among ε4 and ε2 carriers [21]. Children carrying ε4ε4 were found to have lower hippocampal fractional anisotropy and thinner temporal and cingulate isthmus cortices, which were associated with poorer executive function and working memory. Similarly, smaller hippocampal volumes and poorer performance on attention tasks were observed in ε2ε4 children [21]. In line with previous findings on the influence of *APOE* phenotypes on brain development and maturation, Knickmeyer et al. reported that infants carrying the ε4 allele aged between 1 and 3 months had lower grey matter volume in lateral and medial temporal, occipitotemporal, frontal, and precuneus regions, as well as greater grey matter volume in posterior parietal, occipital, middle cingulate, and other frontal and precuneus regions [54].

The relationship between *APOE* ε4 and altered microstructure in the parahippocampal cingulum bundle (PHCB), a neural pathway connecting the posteromedial cortex and medial temporal lobe, has been linked to increased AD risk, notably lower FA and higher mean diffusivity (MD). However, the mechanisms underlying this relationship and their contribution to AD pathology remain unclear. Hodgetts et al. reported an unexpected pattern of higher FA and lower MD in the PHCB of healthy young ε4 carriers aged 18–26 years, suggesting that this hyper-connectivity may increase vulnerability to amyloid-β accumulation and/or tau spread in later life [55]. However, a recent study by Lissaman et al. replicated Hodgetts et al.’s findings but failed to find statistically significant effects in the expected direction and even provided evidence against their presence [56]. Possible explanations for the discrepancy include false positives in Hodgetts et al.’s study or an exaggerated effect size due to a small sample size (15/100 subjects). Although Hodgetts et al.’s study remains relevant and informative, Lissaman et al.’s findings suggest no clear evidence to support the notion that *APOE* ε4-related increases in structural connectivity between posteromedial cortex and medial temporal lobe enhance vulnerability to amyloid-β accumulation and/or tau spread. These findings emphasise the importance of considering the role of *APOE* isoforms in neurodevelopmental processes in healthy subjects when discussing the influence of genetic factors on AD risk in early life. However, the border relevance of these findings in early life remains poorly understood.

#### AD polygenic risk scores

Previous studies have shown that a high AD-PRS may influence mainly the hippocampus volume and, to a lesser extent, the morphometry of other brain regions [45, 50, 57]. Specifically, some structural and functional neuroimaging studies suggest that brain development early in life could be influenced by several genetic variants across the genome, each contributing to different extents to a higher risk of AD.

Although numerous studies have examined the relationship between genetic susceptibility to AD and hippocampal volume, findings have been inconsistent. A study by Walhovd et al. found that higher AD-PRS and carrying the *APOE* ε4 allele were associated with smaller hippocampal volume from ages 25 up to 80 in healthy individuals [57]. However, the AD-PRS effect was not equally associated at all ages among older adults, suggesting possible age-related differences in the effects of genetic risk [57]. Similarly, a study of cognitively healthy young subjects found that higher AD-PRS was associated with smaller hippocampal volume [50]. Consistent with these findings, Axelrud et al. observed a negative correlation between AD-PRS and hippocampal volume and further identified the right CA4 and dentate gyrus as the subregions most strongly associated with AD-PRS in Brazilian samples [45]. Nonetheless, the findings of these studies highlight the complexity of the relationship between genetic susceptibility to AD and hippocampal volume. This association seems to be stronger in studies during early adulthood than during childhood, suggesting that the genetic burden for AD becomes more relevant with age and that there are cumulative processes at play, which may only become apparent after early life.

Axelrud et al. found that increased connectivity between two regions vulnerable to tau pathology, the right precuneus and the right superior temporal gyrus, was associated with inhibitory control and could be impacted during childhood and adolescence [33, 45]. These regions have also been proposed as moderators of the relationship between AD-PRS and memory. Similar findings were observed in young, healthy adults for AD-PRS and hippocampal volume. Foley et al. reported decreased left hippocampal volume and other limbic and paralimbic regions in healthy young ε4 carriers aged 18–32 years [58]. This association persisted even after excluding the *APOE* region from the PRS, indicating that the genetic risk from variants across the genome is not specific to late-life processes. In contrast, other studies did not find a significant effect of the *APOE* ε4 allele on hippocampal volume in young, healthy adolescents [33, 45, 51, 59–61]. Taken together, these results suggest that the development of the hippocampus may be influenced by the cumulative effect of common AD-associated genetic variants that affect cognition across an individual’s lifespan.

The association between genetic risk for AD in early life and cortical thickness is unclear. For instance, while some studies have reported a significant association between the entorhinal cortex (ERC) volume and AD genetic risk, others did not observe an association between the ERC and parahippocampal gyrus (PHG) thickness and AD genetic risk in young, healthy adults [62, 63]. Foley et al. found that a high AD-PRS is associated with a reduction in fractional anisotropy (FA) in the right cingulum bundle, which could indicate white matter damage [58]. Additionally, Essers et al. identified a possible modifying effect of *APOE* status and the AD-PRS on the association between higher air pollution exposure during pregnancy and preadolescence and changes in subcortical and cortical morphology [64]. These findings suggest that genetic susceptibility to AD in early life could influence brain structure under specific circumstances or is unlikely to be clinically relevant. However, further research is needed to clarify the precise nature of this association.

Overall, the relationship between AD risk and structural and functional differences in brain structures is complex and may involve genetic effects on brain structures known to be implicated in the early stages of AD neuropathology. Although some studies have reported an association between AD genetic risk and differences in brain structures, including the hippocampus, entorhinal cortex, and parahippocampal gyrus, evidence suggests that the *APOE* genomic region does not solely drive the effects of genetic risk for AD on brain structures. However, there is still a need for more neuroimaging studies that investigate the relationship between high genetic susceptibility to AD, functional activity and connectivity in the brain, and structural changes in cortical and subcortical structures early in development. Currently, the number of studies examining healthy individuals at different ages still needs to be increased, and future research should aim to fill this gap.

### Relationship between AD genetic risk and early life environment

There is a growing interest in advancing our understanding of which environmental factors maximise the impact of AD-associated genetic variants’ risk.

The impact of genetic factors on cognitive decline is complex and can be influenced by early-life environmental factors. Several studies have explored the impact of social environment, lifestyle, family structure, family/cultural behaviours, and socioeconomic status (SES) in early childhood on cognitive function in late life. For instance, Melrose et al. found that lower childhood SES was associated with a higher rate of global cognitive decline later in life, irrespective of *APOE* genotype in a multiethnic sample [65, 66]. On the other hand, some cross-sectional studies suggest that higher SES in early life is associated with a lower risk of developing AD in late life [67, 68]. However, prospective studies by Wilson et al. [69] and Everson-Rose et al. [69, 70] did not find a significant relationship between early-life SES, cognitive decline, and AD risk in old age. Despite the valuable insights these studies provide on the link between early-life environmental factors, genetic susceptibility to AD, and cognitive decline in late life, further research is needed to fully understand the complex relationships between these variables. A deeper understanding of these relationships can inform interventions and strategies to reduce the risk of cognitive decline and AD in later life.

Air pollution exposure has also been linked to cortical and subcortical brain structure changes due to neuroinflammation and oxidative stress in children and pre-adolescents [64, 71, 72]. Exposure to two pollutants, PMcoarse and polycyclic aromatic hydrocarbons (PAHs), has been associated with alterations in gene expression in the brain [73] and higher incidence of neurodevelopmental disorders [74]. Recent studies have suggested that genetic modifiers, such as *APOE* status and AD-PRS, may modify this association. For instance, Alemany et al. found that higher exposure to PAHs and nitrogen dioxide (NO_2_) is associated with smaller caudate among ε4 carrier children [74, 75]. In contrast, Essers et al. reported that exposure to PMcoarse during pregnancy and PAHs during childhood was linked to larger cerebral white matter volume in *APOE* ε4 carriers than non-carriers. In addition, PMcoarse exposure during pregnancy was associated with larger cortical grey matter volume in children with higher AD-PRS (including *APOE*) [64]. These findings suggest that genetic modifiers may protect neurodevelopment in early life against air pollution exposure and its effects on brain structure. However, the long-term neurodegenerative impacts of air pollution exposure may become more apparent later in life, highlighting the need for further research on the role of genetic modifiers in neurodevelopment.

Recent research has shed light on the potential role of the *APOE* genotype in shaping lipid profiles during childhood and its potential implications for long-term health outcomes. In particular, children with the ε2 allele have been found to have lower cholesterol and higher triglyceride levels than ε3 carriers, while ε4 carriers had elevated levels of both [27]. Consistently, Kallio et al. reported a higher cholesterol and low-density lipoprotein cholesterol (LDL-c) concentration in cord blood from *APOE* ε4 compared to ε2 carriers [27, 76]. A recent study has also reported an association between higher LDL-c levels and PRS-AD in children ε4 carriers, suggesting the *APOE* genotype might affect lipid metabolism in early life, with potential implications for long-term health outcomes [51]. These findings highlight the need for further research to fully understand the relationship between *APOE* genotype, PRS, and lipid metabolism, particularly considering the growing evidence linking dyslipidaemia to AD development.

While positive associations have been identified between several cardiometabolic risk factors in midlife and AD, with higher cardiac function [77] and body mass index (BMI) [78], independently of the *APOE* genotype, the mechanisms by which cardiovascular risk factors may mitigate the risk of AD in those at high polygenic risk have not been thoroughly described [79–81]. Only one study has reported a weak association between AD-PRS and some cardiometabolic risk factors, such as height, lean mass, triglycerides, insulin, and C-reactive protein during childhood and adolescence, indicating that the associations with PRS might emerge later in life [82]. Further research is necessary to elucidate the potential interplay between *APOE* genotype, PRS, cardiovascular risk factors, and AD risk.

Genetic variation in the *APOE* locus within several populations revealed nuanced patterns in genotype distribution, allele frequencies, and heterozygosities. Global investigation of *APOE* allele frequencies in 299 populations revealed significant genetic and geographic variability. Oceania exhibited the lowest frequencies of the *APOE* ε3 allele, while the Indian population showed the highest average frequency. There were apparent differences amongst African populations, with Oceania and Africa having the most genetic diversity. Interesting aspects, such as the absence or low prevalence of the ε2 allele in specific populations and the different distributions of the ε3 and ε4 alleles, were brought to light by the latitudinal variety of *APOE* allele frequencies at different levels [83]. Sigh et al.’s analysis showed that the *APOE* allele frequencies had different clines. For example, the ε4 allele was found to have a consistent north-to-south cline in Europe but not in other regions [83]. These results highlight the intricate interplay among genetic drift, location, and historical selective pressure in modulating APOE polymorphism. Yet, it is crucial to consider that similar differences may arise for other genetic risk alleles linked to NDD. These variations may contribute to differences in prevalence rates and clinical manifestations of these diseases. Unfortunately, evidence in this area is dearth and more research is needed to address these gaps in knowledge.

## Parkinson’s disease

PD is the second most common NDD globally, after AD [84]. Diagnosis of PD is typically made through a clinical examination that covers motor symptoms (MS) and non-motor symptoms (NMS) [85]. MS include resting tremors, bradykinesia and rigidity, whereas NMS may include cognitive decline, depression, and the loss of the sense of smell [86].

### PD-associated genetic factors in early life

Although specific genetic mutations have been associated with familial PD, most cases are sporadic and have an unclear aetiology. Recent studies have identified over 20 genes linked with familial PD and around 90 genetic variants associated with sporadic PD risk [87]. Most studies on PD-associated polygenic risk scores (PD-PRS) have focused on disease risk, age of onset, motor development, and cognitive decline [88].

Only one recent PRS study has examined the relationship between genetic burden for late-life NDDs, including PD, and childhood non-verbal IQ, educational attainment, internalising behaviour, global brain structure, and disease-specific regional brain structures. This study found no evidence that the genetic burden for PD affects the same processes during early life [51], suggesting that the genes implicated in PD aetiology might not be related to cognitive function. It is essential to consider other factors that may impact overburdened genetic differences in susceptibility to PD. However, further studies are needed to evaluate the genetic burden of PD in healthy individuals at an early age before the onset of clinical Parkinson’s symptoms to draw conclusions.

Although brain injuries in early life have been identified as a risk factor for PD later in life [89], it is still unclear whether any structural or functional brain changes might occur due to genetic factors in early life. The structural and functional changes in the brain associated with PD are believed to result from a complex interplay between age, environmental factors, and genetic susceptibility [90]. However, the exact mechanisms underlying PD pathogenesis remain largely unknown.

As the early stages of life are critical for brain development, both genetic and non-genetic factors may influence the future onset of PD. Although the impact of specific PD-associated genetic variants on brain structure and morphology related to PD development and progression has been widely studied [91], only one study has evaluated the association between PD-PRS and the rate of changes in the brain in a cohort of healthy children aged 9–12 years. This study failed to establish a significant association between the genetic burden of PD and changes in brain volume [51]. This inconsistency may be due to ethnic and age-related differences among the participants. Therefore, it is crucial to investigate the relationship between PD genetic burden and changes in brain morphology and function in health during early life, including racially diverse samples.

### PD-associated non-genetic factors in early life

The interplay of age, environmental factors, and genetic susceptibility is believed to play a role in the development of PD [90]. Other non-genetic factors such as educational attainment, IQ, handedness, sociodemographic factors, and lifestyle variables such as caffeine intake, iron intake, alcohol consumption, smoking, and postmenopausal hormones are also considered relevant.

PD has been associated with higher education levels and occupations that require more education [92, 93], unlike AD, which has been linked to lower education levels, IQ, and SES [94, 95]. One study conducted in Sweden found that high cognitive capacity, as measured by IQ scores, might be associated with an increased risk of developing PD later in life [96]. Interestingly, the same study proposed that smoking, associated with reduced risk for PD, may be linked to high IQ. However, further research is required to validate this connection [96].

The relationship between smoking and PD has been widely discussed, with some studies suggesting that nicotine and other compounds in cigarette smoke have a cytoprotective effect [97–100]. Fardell et al. observed a negative correlation between smoking and the development of PD and IQ [96]. However, conflicting findings have also been reported, with some studies identifying cytoprotection as a potential causal factor for PD [101, 102]. The cytoprotective properties of nicotine are thought to be mediated by its ability to activate downstream processes that reduce neuronal damage through the enzyme SIRT6. Although SIRT6 is beneficial for cancer cells, its activation may lead to neuronal death and the development of PD. Nicotine has been shown to decrease SIRT6 levels, which may provide neuroprotection [103].

Recent research has suggested that a higher IQ in early life could be a potential risk factor for developing PD later in life. In contrast, alcohol and caffeine consumption might have a protective effect [87, 104–107]. A large prospective study on healthy middle-aged individuals showed a robust and highly significant inverse relationship between caffeine consumption and PD risk [104]. Similar findings were observed in case-control studies from various cohorts, including Germany and Sweden [105], Japan-America [106], and Rochester [107], suggesting that moderate and chronic caffeine intake might reduce the PD risk in both healthy individuals without a history of dementia and PD cases. Caffeine is an antagonist to adenosine A2A (*ADORA2A* gene), leading to increased dopamine neurotransmission and reduced PD risk [108]. Ascherio et al. investigated the relationship between caffeine intake and PD risk, revealing a gender difference in the association [104]. Men who consume caffeine were found to have a lower risk of developing PD, while the association was U-shaped for women, with those consuming moderate levels of caffeine observed to have the lowest risk of PD [104]. Further investigation was carried out to understand if the use of hormones after menopause affects the association between caffeine intake and PD risk in women.

Oestrogen levels may influence the risk of PD associated with caffeine consumption in women. However, it is still unclear if the neuroprotective ability of oestrogen is the reason for the overall lower risk of PD in women compared to men [109]. Additionally, Ascherio et al. revealed a significant correlation between other cognitive functions, such as verbal, visuospatial, and technical abilities, and the risk of PD, suggesting that cognitive capacity may contribute to the disease [96]. These findings highlight the need for further research on the complex relationship between various factors and PD risk.

#### Future perspectives

PRS research has attracted significant attention due to its ability to identify individuals at high risk for developing NDDs, based on their genetic susceptibility years before clinical symptoms appear. Recent advances in genomics and neuroimaging have facilitated a better understanding of the genetic factors involved in NDDs and their impact on cognitive function and brain structure from an early age. PRS has the potential to revolutionise our understanding of these complex polygenic forms of NDDs by aiding in screening individuals at higher risk for disease based on their genetic profile. However, significant challenges must be addressed to ensure the accuracy and applicability of PRS.

The accuracy of PRS is influenced by various factors, including the statistical power of the original GWAS, genetic variants considered, and demographic variables such as ethnicity and gender. Selecting an appropriate p-value threshold from GWAS summary statistics is critical in building PRS. However, the selection criteria for this threshold vary according to the research question being addressed, leading to significant heterogeneity in PRS due to differences in the calculation process. For instance, Mormino et al. revealed a relationship between AD-PRS and AD-like ß-amyloid protein levels in healthy younger individuals using SNPs below the standard GWAS p-value threshold (*p* = 5 × 10^−08^) [50]. This variability remains a significant obstacle to the clinical application of NDD-PRS, and further research is necessary to develop standardised criteria for selecting p-value thresholds in PRS studies.

Combining *APOE* haplotypes and PRS with other risk factors could lead to more accurate AD risk prediction. However, much is still debated about how AD genetic burden affects cognitive health in non-dementia patients during early life. Although some research suggests that the association between *APOE* and cognitive decline might have roots in early life, caution must be exercised in interpreting causal relationships as various factors such as ethnicity, age, sex, and the type of cognitive assessment employed can mitigate the outcome. Additionally, the estimation of PRS can be biased by differences in cognitive measures, leading to inconsistencies in findings across previous studies considered in this review. One critical consideration is understanding the accuracy of AD-PRS when excluding the *APOE* ε4 allele risk in various healthy cohorts at different ages.

On the other hand, the development of large-scale GWAS studies and data-sharing policies have led to the optimisation and updating of PRS studies to draw robust and informative conclusions about NDDs. Previous studies have suggested that a high AD-PRS may primarily affect the hippocampal volume and, to a lesser extent, the morphometry of other brain regions in early life among healthy cohorts. However, inconsistencies in findings suggest that other factors, such as sample size and availability of neuroimaging data from healthy cohorts of different ages, may influence the relationship between genetic susceptibility to AD and hippocampal volume.

Future research should prioritise the development of effective preventive and therapeutic strategies for NDDs, considering the complex interplay of genetic, environmental, and lifestyle factors. As most PD cases have an unclear aetiology, further studies are needed to investigate the relationship between PD genetic burden and other non-genetic factors in healthy individuals during early life before the onset of clinical Parkinson’s symptoms.

The application of PRS in the field of NDDs is crucial for drug target discovery and accurate and robust treatment based on pharmacogenetic variants. In the same vein, it would be relevant to consider the potential ethical implications of using PRS for risk prediction and how this information may be used in clinical decision-making. Investing in healthy cohort research and addressing key challenges can lead to significant advancements in detecting several NDDs. Such progress can improve the lives of individuals at risk of developing NDDs and their families.

## Conclusions

In conclusion, the ability of PRS to predict the onset of NDDs may allow for the early detection of these conditions. It may help identify people more likely to develop certain diseases based on their genetic profile. However, significant challenges must be addressed, such as the low availability of samples from various cohorts and the high variability of samples. Despite these obstacles, the growth of more extensive GWAS studies and data-sharing regulations encourages the optimisation and updating of NDDs-PRS for making explicit judgments on various NDDs. Future research should prioritise the development of effective preventive and therapeutic strategies for NDDs, considering the complex interplay of genetic, environmental, and lifestyle factors. Investing in healthy cohort research and addressing key challenges can lead to significant advancements in detecting several NDDs, improving the lives of individuals at risk of developing NDDs and their families.
